# Successful model of shared medical directorship in Hospital Epidemiology

**DOI:** 10.1017/ice.2023.228

**Published:** 2024-03

**Authors:** Christina Liscynesky, Shandra R. Day, Nora E. Colburn, Susan L. Koletar

**Affiliations:** Division of Infectious Diseases, Wexner Medical Center, The Ohio State University, Columbus, Ohio

*To the Editor—*Healthcare Epidemiology programs are a requirement of the Joint Commission and the Centers for Medicare & Medicaid Services.^1,2^ The nationally recommended ratio for infection preventionists (IPs) is 1 IP per 69 inpatient beds.^3^ The amount of necessary physician support is less clear. Should it be delineated based on inpatient bed number, ambulatory clinic numbers, or geographic area?

Traditionally, a single medical director oversees the department of Hospital epidemiology, and depending on the size and needs of the institution, associate medical directors (AMDs) may be needed as well. We report our successes in challenging that classic framework with a co–medical-director model.

## Background

The Ohio State University Wexner Medical Center is an academic, quaternary health system with ∼1,600 beds, comprising University Hospital (Level 1 trauma/burn center/liver & kidney transplant), the James Cancer Hospital (bone marrow transplant unit), Ross Heart Hospital (heart and lung transplant), East Hospital (orthopedic surgery) as well as ∼200 clinics including 4 ambulatory surgery centers.

## Creation of co–medical-director model

The hospital epidemiology department historically had 1 medical director until 2012, when an AMD was added to provide oversight to the James Cancer Hospital. In 2014, another AMD was recruited with oversight at East Hospital. The medical director fielded emergency calls from IPs and leadership 24/7, presented at leadership forums, and approved all decisions. The enormous job scope left minimal opportunity for preparedness or clinical work.

In January 2018, the longtime medical director retired, providing the opportunity to reassess the traditional top–down leadership model. The AMDs worked as a team to identify operational needs, for example, starting the Influenza Workgroup. For the next 18 months, we continued with our primary epidemiology responsibilities and split the remaining responsibilities based on clinical obligations; the hospital leadership responded with supplemental pay. An added benefit was seamless coverage for each other while on leave (vacation or medical). Efficiency improved because approval and guidance was only needed from 1 physician, which allowed real-time decision making. The dyad leadership success built upon interpersonal trust, constant communication, meticulous organizational skills, and task delegation to the IPs. Our IPs grew in their roles as experts as we empowered them to make decisions. We created protocols for common exposures such as Norwegian scabies, led outbreak investigations for pathogens including group A *Streptococcus*, and created institutional guidance for emerging pathogens such as Ebola. The benefits of excellent team communication, increased availability for collaboration, and preplanning were quickly realized and acknowledged with increased institutional support. In August 2019, the Ross Heart Hospital funded a third AMD.

## Experience during the COVID-19 pandemic

As hospital epidemiologists, we were at the center of the medical center’s response to the global crisis. We collectively led the COVID-19 Clinical Care Workgroup (CCWG), a multidisciplinary team responsible for protocol and guideline creation. In the setting of a new virus, compounded by panic from the press, family and coworkers, this amount of work was clearly untenable for 1 person. Our prior transition to a shared leadership model facilitated quick group decision making. Individual strengths and differences (ie, personalities, experiences, and risk tolerances) allowed us to challenge each other on policies prior to roll out. This model allowed us to remain active on the infectious diseases (ID) consultation services, providing us frontline experience to ensure practical polices as we literally went from the bedside to the boardroom for daily meetings with leadership. Importantly, we took scheduled breaks, including vacations and medical and maternity leave to avoid burnout. Our team has come out of the pandemic stronger, more cohesive, and with the respect of hospital leadership. The 3 AMD titles have been transitioned to medical director titles, underscoring the triumvirate leadership mode.

## Current organization

**Figure 1.**
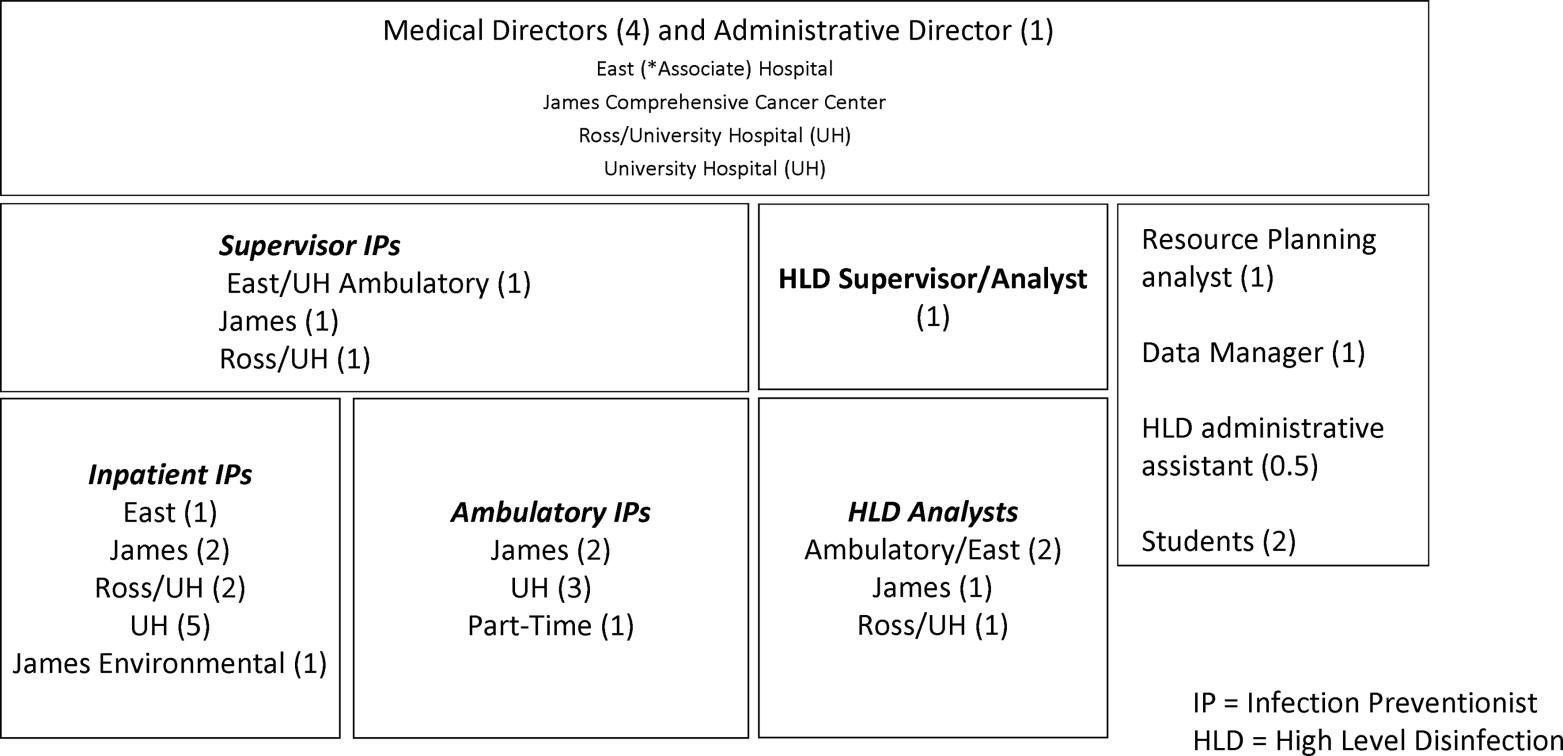
Epidemiology department.

The current department is composed of 3 medical directors; 1 associate medical director; 1 administrative director; and 20 IPs with diverse subject-matter expertise, including 5.5 IPs dedicated to ambulatory, 1 data manager, 1 planning analyst, and 5 high-level disinfection analysts (Fig. 1). We built a collaborative, dynamic department that functions at its peak performance regardless of which medical director is available. The medical directors each have salary support of ∼0.5 full-time equivalent (FTE), with the associate medical director at 0.35 FTE.

## Discussion

A key component to our success is that it allows for continued patient care, providing balance, clarity, and the privilege of managing interesting ID cases. The consultation service allows us to experience the environment we are safeguarding, to interact with the frontline staff, and to teach our trainees at the bedside and as rising healthcare leaders.^4^

Patient care is not interrupted with administrative questions because an off-service medical director is available. This flexibility advances infection control initiatives by not delaying meetings—for example, rapid institutional rollout of new *Clostridiodes difficile* testing and permanent presence on device-related infection rounds. The biggest challenge to this model is minimizing work duplication and ensuring efficient use of everyone’s time. Efficiency requires constant communication to ensure that we are abreast of current issues and that we prioritize the determination of who is leading an initiative. Having a systemwide administrative director is key to maintaining this balance and providing consistent communication across the health system. We are also very conscientious about the challenge of providing conflicting guidance, and we work closely to provide a clear and unified message.

In conclusion, the creation of a hospital epidemiology co–medical-director model has allowed for a sustainable program able to withstand the challenges of the pandemic while allowing time to balance personal and professional goals. The key to the success of our model is continued communication and staunch institutional support.
